# Early Maturation of Heart Rate Variability in Very Preterm Infants Depends on Neonatal Factors and Is Associated With Neurodevelopmental Risk

**DOI:** 10.1111/apa.70462

**Published:** 2026-02-12

**Authors:** Léa Bonneau, Cyril Flamant, Maxime Esvan, Jean Michel Roué, Géraldine Favrais, Géraldine Gascoin, Sandie Cabon, Fabienne Porée, Guy Carrault, Patrick Pladys

**Affiliations:** ^1^ Department of Paediatrics Rennes University Hospital Rennes France; ^2^ Univ Rennes, Inserm Rennes France; ^3^ Department of Neonatology Nantes University Hospital Nantes France; ^4^ Clinical Pharmacology Department, Center of Clinical Investigation, Inserm Rennes University Hospital Rennes France; ^5^ Department of Neonatology Brest University Hospital Brest France; ^6^ Department of Neonatology Tours University Hospital Tours France; ^7^ Department of Neonatology Angers University Hospital Angers France

**Keywords:** autonomic nervous system, biomarker, heart rate variability, neurodevelopment, prematurity

## Abstract

**Aim:**

This study investigated central autonomic network maturation deviations using a previously defined machine learning model set to estimate a functional maturation age (FMA) from heart rate variability (HRV) analysis. We investigated whether these deviations were associated with maternal, fetal, perinatal, or postnatal complications and with neurodevelopment at 2 years of age.

**Methods:**

Differences (ΔHRV) between post‐menstrual age (PMA) and FMA were measured in a multicenter prospective cohort of 132 preterm infants born < 30 weeks gestational age (GA). The relationship between ΔHRV and clinical factors, occurrence of complications or alterations in neurodevelopment assessed by Ages and Stages Questionnaire (ASQ) at 2 years of age was studied.

**Results:**

ΔHRV expressed in weeks delay at 34 weeks of PMA was greater when GA was lower (3 IQR 2.3–3.9) at 24–26 versus 1.3 (IQR 0.8–2.4) at 28–30 weeks GA (*p* < 0.0001), in cases of bronchopulmonary dysplasia (*p* < 0.0001) or patent ductus arteriosus (*p* < 0.0001). ΔHRV was also associated with altered social skills at 2 years of age (OR 2.05, 95% CI 1.02; 4.14 for each week, *p* < 0.05) but not with ASQ total score (*p* = 0.06).

**Conclusion:**

ΔHRV, early and non‐invasively estimated, depends on GA, perinatal and postnatal complications. Its assessment could contribute to evaluation of neurodevelopmental risk.

## Summary

- Heart rate variability in very preterm infants provides a functional estimation of central autonomic network maturation.
- Using a machine learning model, the study highlights and quantifies the impact of gestational age, bronchopulmonary dysplasia, and patent ductus arteriosus on delayed autonomic maturation.
- This delay in autonomous maturation is associated with subsequent developmental delay at the age of two, suggesting the possibility of early identification of abnormal maturation trajectories.

**Abbreviations**
95% CI: 95% confidence interval
ΔHRV: difference between functional maturation age and post‐menstrual age expressed in weeks
ASQ: Ages and Stages Questionnaire, third edition
BPD: bronchopulmonary dysplasia
FMA: functional maturation age
GA: gestational age
HRV: heart rate variability
IQR: interquartile range
ORs: odds ratio
PDA: patent ductus arteriosus
PMA: post‐menstrual age

## 1 Introduction

Preterm birth is a one‐time event that can have severe lifelong medical consequences. Morbidity associated with prematurity is inversely proportional to gestational age (GA), with an increased risk when major events occur during hospitalisation [1, 2, 3]. The first 2 years of life, marked by brain plasticity, have been identified as a critical window for personalised interventions to prevent neurodevelopmental abnormalities. This justifies the search for early biomarkers of neurodevelopmental risk.

The evaluation of neurological maturation and neurodevelopmental outcome risk is complex and can vary widely among neonatologists [4]. Predictive statistical models and machine learning models most often incorporated several types of variables. The variables used in the models included clinical data on perinatal events, neurobehavioural assessments, socio‐economic data, and brain imaging or electroencephalogram results [5, 6, 7, 8]. These models improved clinical evaluation, but were limited in terms of precision and clinical usefulness. This limitation was mainly due to the heterogeneity of variable definitions, the lack of standardisation of results, and the intrinsic limitations of the analytical methods used [9].

Heart rate variability (HRV) reflects interactions between the autonomic and central nervous systems via the central autonomic network [10, 11, 12]. HRV is known to be influenced by GA [13, 14] and nervous system maturation [15, 16]. HRV analyses have been proposed to develop models for monitoring clinical status throughout the perinatal period and predicting neurodevelopmental outcome [17, 18, 19, 20]. Since HRV measurements are easily accessible at the bedside, they offer a promising tool for tracking functional neurological maturation and aiding neurodevelopmental assessment.

HRV maturation has been quantified via ensemble machine learning or Gaussian process regression modelling in two publications published in 2022–2023 [15, 16]. In those studies, HRV functional maturation age (FMA) was correlated with postmenstrual age (PMA), with mean absolute errors of 1.7 and 0.9 weeks, respectively [15, 25]. FMA has also been correlated with the infant's functional brain age, an index of cerebral maturation derived from an electroencephalogram [8, 15]. Using these methods, it is now possible to quantify ΔHRV, defined as the difference between FMA estimated by machine learning and the PMA of the preterm infant. This quantification of ΔHRV allows for an estimation of the delay in maturation of the autonomic nervous system. In this study, we used a machine learning ensemble method to measure ΔHRV. The objective was to identify clinical factors associated with increased ΔHRV. We also examined whether delayed maturation as measured by ΔHRV was associated with neurodevelopmental disorders at corrected age two, as assessed using the Ages and Stages Questionnaire (ASQ).

## 2 Methods

### 2.1 Cohort Details

Data for this study were extracted from the Digi‐NewB observational cohort database (NCT02863978, Horizon 2020 European project GA‐689260). The Digi‐NewB project aimed to improve neonatal care by developing a noninvasive perinatal health monitoring system based on artificial intelligence analysis of clinically relevant functions. Data were prospectively collected from more than 600 newborn infants born at 25–42 weeks of PMA in six French university hospitals between 2017 and 2020. A subset of this cohort was dedicated to study different aspects of neonatal maturation included the current study on HRV maturation. The current sub‐study considered preterm infants born before 30 weeks of gestational age with available ECG data. All preterm infants who had not been used in the previous study aimed at training the model were included [16].

The Newborn Individualised Developmental Care and Assessment Program [21] was implemented in all participating centres. All infants included underwent individualised and systematised multi‐professional assessment and intervention up to the age of two.

### 2.2 Variables Studied

The data were collected from the medical records of the newborn infants and their mothers during their hospitalisation in neonatal units. Maternal and prenatal variables included maternal age at birth, active smoking during pregnancy, and variables related to pregnancy complications. The definition of active smoking during pregnancy was based on the mother's declaration; no minimum frequency was needed. The complications considered were maternal arterial hypertension, gestational or type 2 diabetes, chorioamnionitis, and premature rupture of the amniotic membrane defined as a rupture > 12 h before delivery. Betamethasone exposure and magnesium exposure were also noted.

The variables related to the birth and the newborn infants included GA at delivery, sex, birth weight, Fenton's percentile of GA, Apgar score at 5 min and any resuscitation techniques in the delivery room. The GA was always confirmed by obstetric ultrasonography. The variables related to neonatal hospitalisation included the presence and classification of structural brain lesions, bronchopulmonary dysplasia (BPD), and significant patent ductus arteriosus (PDA). BPD was defined as the need for ventilatory support or oxygen requirements at 36 weeks of PMA [22]. PDA was diagnosed by transthoracic ultrasound, which was a routine practice in all the centres [23]. The assessment of significant PDA was based on confrontation of clinical and echocardiographic criteria following the American Academy of Paediatrics Committee on Fetus and Newborn, 2014–2015 [23]. The neurological lesions were classified as grade 1–4 intraventricular haemorrhage [24], noncystic or cystic white matter injury, or other neurological lesions detected on ultrasound or MRI. Late‐onset sepsis was deemed to be present in preterm infants with suspected sepsis at least after 3 days of life and who received more than 5 days of antibiotic therapy.

### 2.3 Proposed Approach for Estimation of HRV Functional Maturation Age

The ensemble machine learning algorithm used to estimate FMA, including the signal processing and extraction of the HRV features, feature selection and generalisation of the proposed model, has already been described in detail [16]. In summary, Leon et al. acquired and processed the raw signals collected from bedside Philips Intellivue monitors (Philips, The Netherlands) during the infant's stay in neonatal units, which was used for HRV detection. To train and test the model, the HRV data were acquired from 50 healthy infants, born between 25 and 41 weeks of GA, who did not present any signs of abnormal maturation relative to their age group. The selection of these infants was performed on the basis of a three‐step clinical evaluation performed by two senior neonatologists. They selected newborn infants on the basis of the absence of congenital or perinatal pathologies and with a clinical evolution throughout the neonatal hospitalisation period that could be considered normal for their GA according to the clinical health reports. The exclusion criteria used to select the newborn infants eligible for inclusion in the reference group were detailed in Table S1. Electrocardiographic (500 Hz) recordings were collected from bedside monitors. They were obtained from birth to discharge, continuously during the first 3 weeks of life and then during a 24‐h period every 10 days until discharge. Cardiac cycle length time series were extracted and segmented into 30‐min periods with good quality traces. Analyses of HRV were performed using several methods: time‐domain, frequency‐domain, and non‐linear analyses. These non‐linear analyses included fractal, entropy, Poincaré plot analyses and visibility graph methods. The selection of HRV features to be used in the model was processed using genetic algorithm [16]. In summary, the method consisted of testing several groupings of 20 variables and then carrying out selection, crossover and mutation operations to generate new populations of solutions. This was done with the objective to converge on an optimal or near‐optimal selection of variables to create the predictive model [16].

**FIGURE 1:**
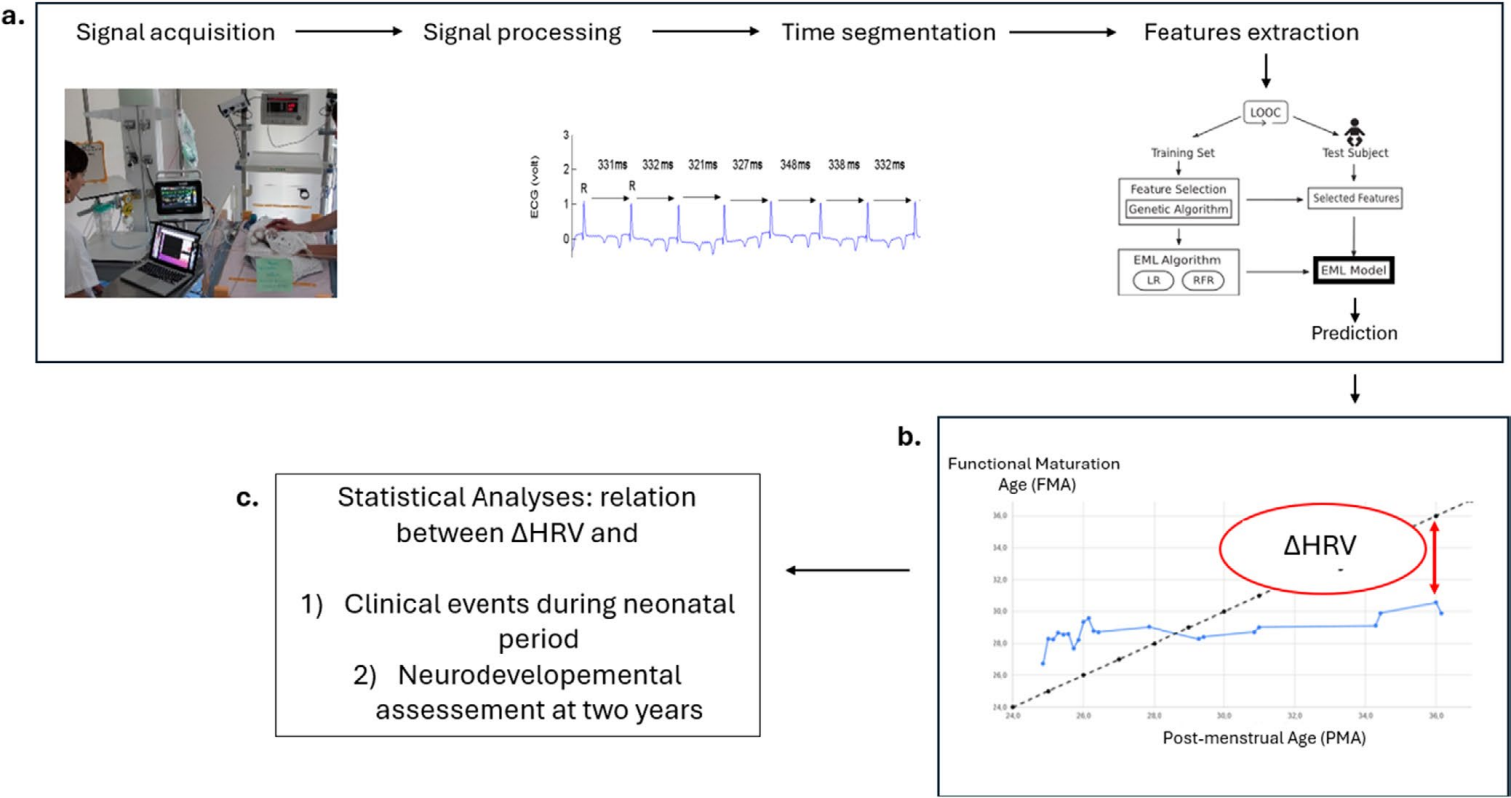
Overview of the study. (a) The whole process for signal processing, HRV feature extraction and selection, and Ensemble Machine Learning used has already been described in detail [16]. (b) ΔHRV was defined as the difference between post‐menstrual age (PMA) and functional autonomic maturation age estimated by machine learning (FMA). The dashed line corresponds to the expected ideal central autonomic network maturation built in the training phase of the study. The observed individual FMA trajectories may have been influenced by prematurity and perinatal events. (c) Using the parental questionnaire Ages and Stages Questionnaire, third edition, we assessed if ΔHRV was related to perinatal events and to neurodevelopmental outcome at the corrected age of 2 years.

We used 15 selected HRV features in conjunction with the GA as input parameters of the FMA estimator. Then the estimator combined logistic and random forest regressions that model linear and non‐linear dynamics between HRV features and PMA. A leave‐one‐out‐cross‐validation technique was used to train and test the model (Figure 1).

### 2.4 Neurodevelopmental Evaluation at the Corrected Age of 2 Years

Neurodevelopmental outcomes at 2 years of corrected age were assessed using the third edition of the Ages and Stages Questionnaire (ASQ). ASQ [25] is a parent‐reported developmental screening tool covering five domains: communication, gross motor, fine motor, problem solving, and personal‐social skills. Items are scored on a three‐point scale: yes (10 points), sometimes (5 points), or not yet (0 points), with a total score range of 0–60 per domain. The questionnaire was sent to parents via email after a phone briefing. Responses were accepted only if completed between 21 and 26 months of corrected age. Scores were used as binary variables, with developmental delay indicated by scores more than two standard deviations below the expected for age (thresholds: 35 for communication, 25 for others). A total score ≤ 220 was considered abnormal [26]. If one or two items were missing per domain, an adjusted score was calculated using the online ASQ tool. Domains with more than two missing items were excluded.

### 2.5 Statistical Analyses

For each patient, outliers in the FMA were eliminated using the formulas first quartile—1.5 × interquartile range (IQR) for low outliers and third quartile + 1.5 × IQR for high outliers. A linear mixed‐effects model was constructed using all available ΔHRV from 32 to 36 weeks of PMA and considered patient as a random effect to account for repeated measures within individuals. The model was adjusted to account for the presence of intrauterine growth retardation, BPD, neurological lesions, and PDA. The GA variable was not included in the model, as it is already considered in the estimation of FMA. The maturation was expressed by FMA, which was interpolated by the linear mixed‐effects model at 34 weeks of PMA. The term 34 weeks of PMA is indicative of the threshold for potential transfer to a local hospital or discharge to a home hospitalisation unit. This value was used in the subsequent analyses as an estimation of the maturation delay during the course of the patient's hospitalisation. Descriptive analysis was performed, with categorical variables expressed as counts and percentages, and quantitative variables as medians and quartiles. Student's *t*‐test, Analysis of Variance or Mann–Whitney–Wilcoxon test compared quantitative variables, while the chi‐square test or Fisher's exact test was used for categorical variables. Binary logistic regression was employed to assess the relationship between neurodevelopment at 2 years in very preterm infants and perinatal clinical features for the ASQ domain associated with ΔHRV. Results are presented as odds ratios (ORs) with 95% confidence intervals (95% CI). Statistical analysis was conducted using SAS, version 9.4 (SAS Inc., North Carolina, USA), with a significance level of 0.05.

### 2.6 Ethics Statement

This restrictive inclusion criterion was used to prevent overfitting of the machine learning algorithm. The study was approved by the ethics committee “Comité de protection des personnes Ouest IV” (34/16) and the National Agency for the Safety of Medicines (Authorization number: 2016062400181), with signed informed parental consent.

## 3 Results

### 3.1 Sample Characteristics

The Digi‐NewB cohort subset dedicated to study neonatal maturation included 379 newborn infants. Among them, 170 were born before 30 weeks of GA, and 132 (77%) of them were included in this study (Table 1). The group of 38 newborns not included comprised 23 newborns who did not have the necessary electrocardiographic recordings and 15 newborns who had participated in the learning phase to build the model. The ASQ questionnaire at 2 years was available for 87 infants (66% of those included). Infants not included had a higher GA at delivery and fewer complications (Table S2), while those who did not complete the ASQ assessment were less mature and had more neurological lesions than those who did (Table S3).

**TABLE 1:** Population characteristics and associations between ΔHRV at 34 weeks of PMA and perinatal characteristics.

	Categories	*N* (%)^a^	ΔHRV^a^	*p*
Maternal and antenatal characteristics				
Maternal age (years)	19–26	28 (21.2)	2.3 (1.7; 3.5)	0.55
	27–31	37 (28.0)	2.3 (1.1; 3.3)	
	32–36	41 (31.1)	1.8 (0.9; 2.8)	
	37–45	26 (19.7)	2.3 (0.9; 2.9)	
Arterial hypertension	No	107 (81.1)	2.3 (1.1; 3.2)	0.27
	Yes	25 (18.9)	2.0 (0.9; 2.6)	
Chorioamnionitis	No	98 (74.2)	2.3 (1.0; 3.0)	0.40
	Yes	34 (25.8)	2.3 (1.2; 3.5)	
Premature rupture of membranes	No	89 (67.4)	2.3 (0.9; 3.0)	0.38
	Yes	43 (32.6)	2.3 (1.3; 3.2)	
Type 2 or gestational diabetes	No	120 (90.9)	2.3 (1.1; 3.0)	0.91
	Yes	12 (9.1)	2.2 (0.9; 3.1)	
Betamethasone exposure	No	7 (5.3)	1.6 (0.9; 3.0)	0.41
	Yes	125 (94.7)	2.3 (1.1; 3.0)	
Magnesium exposure	No	26 (19.7)	1.9 (1.1; 3.0)	0.36
	Yes	106 (80.3)	2.3 (1.0; 3.0)	
Tobacco	No	118 (89.4)	2.3 (0.9; 3.0)	0.12
	Yes	14 (10.6)	2.5 (2.3; 3.4)	
Birth characteristics				
Gestational age (weeks)	< 26	31 (23.5)	3.0 (2.3; 3.9)	< 0.001
	26–28	52 (39.4)	2.3 (1.3; 3.0)	
	≥ 28	49 (37.1)	1.3 (0.8; 2.4)	
Infant sex	Female	64 (48.5)	2.3 (0.9; 3.3)	0.29
	Male	68 (51.5)	2.3 (1.1; 2.9)	
Weight (percentile for gestational age)	< 25	33 (25.0)	2.3 (0.9; 3.0)	0.84
	25–50	27 (20.5)	2.3 (0.9; 3.3)	
	50–75	41 (31.1)	2.3 (1.1; 2.8)	
	≥ 75	31 (23.4)	2.3 (1.1; 3.4)	
Apgar score at 5 min^b^	< 7	30 (23.3)	2.9 (1.8; 3.4)	< 0.05
	≥ 7	99 (76.7)	2.3 (0.9; 3.0)	
Ventilation	No	6 (4.5)	1.0 (0.1; 1.2)	< 0.05
	Yes	126 (95.5)	2.3 (1.1; 3.0)	
Chest compression	No	122 (92.4)	2.3 (1.1; 3.0)	0.18
	Yes	10 (7.6)	3.1 (0.9; 4.1)	
Complication during hospitalisation				
Late onset sepsis	No	41 (31.1)	2.3 (1.3; 3.2)	0.39
	Yes	91 (68.9)	2.3 (0.9; 3.0)	
Necrotizing entorocolitis	No	114 (86.4)	2.3 (0.9; 3.0)	0.25
	Yes	18 (13.6)	2.3 (1.8; 3.2)	
Neurological lesions	No	95 (72.0)	2.3 (0.9; 2.9)	0.20
	Yes	37 (28.0)	2.4 (1.2; 3.6)	
Neurological lesion types	IVH grade 2	15 (11.4)	2.3 (0.8; 3.6)	0.07
	IVH grade 3–4	6 (4.5)	3.7 (2.3; 4.8)	
	Noncystic leukomalacia	5 (3.8)	2.3 (2.3; 2.4)	
	Cystic leukomalacia	5 (3.8)	2.0 (0.9; 2.3)	
	Other neurological lesion^c^	6 (4.5)	3.2 (2.8; 3.8)	
Bronchopulmonary dysplasia	No	87 (66.0)	2.2 (0.9; 2.4)	< 0.001
	Yes	45 (34.0)	3.0 (2.0; 3.7)	
Patent ductus arteriosus	No	46 (34.8)	1.1 (0.8; 1.6)	< 0.001
	Yes	86 (65.2)	2.6 (2.3; 3.5)	

*Note:* ΔHRV (expressed in weeks) quantify the delay in maturation as measured by the difference between post‐menstrual age and functional maturation age, the latter being estimated by the machine learning model defined from the analysis of heart rate variability (HRV).

^a^ Data are presented as *N* (%) for categorical variables and median (first quartile; third quartile) for ΔHRV at 34 weeks gestational age (GA); Students *t*‐test, analysis of variance or Mann–Whitney–Wilcoxon test compared quantitative variables, while the chi‐square test or Fishers exact test was used for categorical variables.

^b^ 3 missing data.

^c^ Other neurological lesions are as follows right frontal schizencephaly and polymicrogyria, septal agenesia, ischemic lesions of lenticular and caudate nuclei and ischemo‐hemorrhagic lesions of vermis and corpus callosum, bilateral parieto‐insular clastic lesions and thin corpus callosum, left cerebellar microhemorrhage, and right subependymal nodular hyperechogenicity.

### 3.2 ΔHRV at 34 Weeks of PMA

**FIGURE 2:**
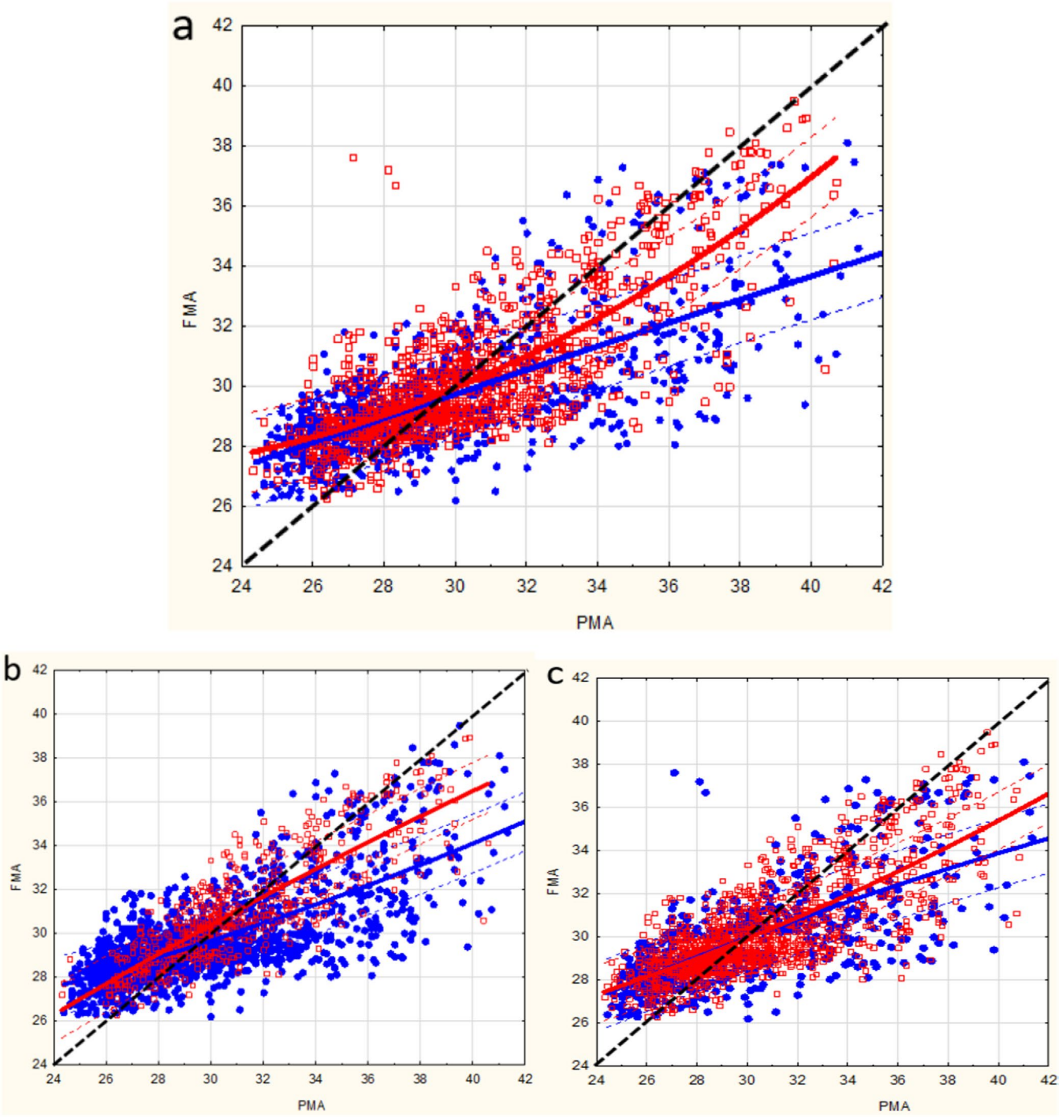
Points represent individual functional maturation age (FMA) estimated from birth to hospital discharge. Lines represent the median FMA. The results are presented according to the presence (blue) or absence (red) of complications: (a) bronchopulmonary dysplasia; (b) patent ductus arteriosus; c. neurological lesions.

The results are presented in Table 1. Notably, early‐onset sepsis has not been presented, as none of the preterm infants included had these variables. A significant increase in ΔHRV measured at 34 weeks of PMA was associated with low GA at delivery (*p* < 0.001), BPD (*p* < 0.001), PDA (p < 0.001), ventilation at birth (*p* < 0.05) and an Apgar score below seven at 5 min (*p* < 0.05). The delay associated with low GA ranged from 3 (IQR 2.3–3.9) weeks < 26 weeks GA to 1.3 (IQR 0.8–2.4) ≥ 28 weeks GA (*p* < 0.001). There was a trend toward increasing ΔHRV at 34 weeks of PMA, depending on the type of neurological lesions among infants with neurological lesions (*p* = 0.07). The maturation trajectories are presented in Figure 2. We observed that the HRV maturation trajectories of the included very preterm infants did not follow the dotted line, which corresponds to the line of identity between FMA and PMA as defined by the machine learning model. We observed a delay in maturation which increased with postnatal age to 2.3 weeks (IQR 1.1–3.0) at 34 weeks of PMA (Figure 2).

### 3.3 ΔHRV and ASQ

**FIGURE 3:**
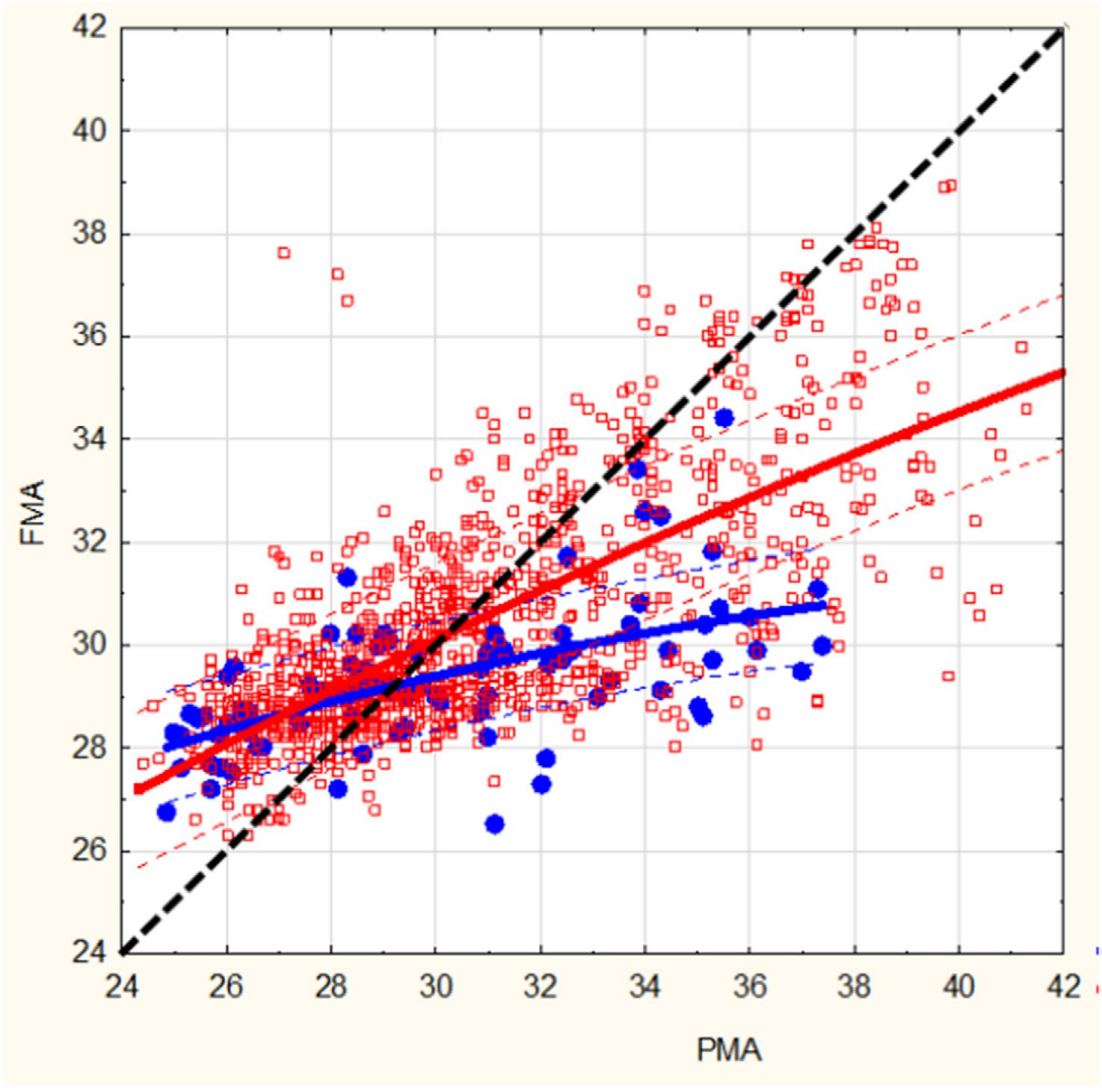
Points represent individual functional maturation age (FMA) estimated from birth to hospital discharge. Lines represent the median FMA. The results are presented based on the results of personal‐social skill assessments (Ages and Stages Questionnaire) at 2 years: In blue for preterm infants with impaired personal‐social skills (score more than two standard deviations below the expected for age) in red for the other preterm infants.

An increase in ΔHRV at 34 weeks of PMA, indicated delayed maturation, was associated with impaired personal‐social skills at 2 years of age (*p* < 0.05) (Table 2). We found no associations with the other neurodevelopmental skills evaluated by the ASQ total score (*p* = 0.06) and the other ASQ sub‐scores. The associations between ΔHRV at 34 weeks of PMA and fine motor skills were not tested because of the absence of infants with a score below the threshold for this domain. The maturation trajectories according to personal‐social skills are presented in Figure 3.

**TABLE 2:** ΔHRV at 34 weeks of PMA according to the ASQ domains (Ages and Stages Questionnaire, third edition).

Variables	ASQ score	*N* (%)^a^	ΔHRV^a^	*p*
Total score^b^	Abnormal	21 (24.7)	1.8 (2.6; 3.5)	0.06
	Normal	64 (75.3)	0.9 (1.9; 3.1)	
Communication	Abnormal	20 (23)	2.4 (1.0; 3.3)	0.42
	Normal	67 (77)	2.3 (0.9; 3.3)	
Gross motor^c^	Abnormal	5 (5.8)	0.9 (0.3; 4.8)	0.95
	Normal	81 (94.2)	2.3 (1.0; 3.3)	
Fine motor^b^	Normal	85 (100)	2.3 (0.9; 3.3)	NA
Problem solving	Abnormal	4 (4.6)	2.0 (1.1; 3.5)	0.76
	Normal	83 (95.4)	2.3 (0.9; 3.3)	
Personal‐social	Abnormal	6 (6.9)	4.0 (1.8; 4.8)	< 0.05
	Normal	81 (93.1)	2.2 (0.9; 3.0)	

Abbreviation: NA, not applicable.

^a^ Data are presented as *N* (%) for categorical variables and median (first quartile; third quartile) for ΔHRV at 34 weeks of PMA; Students *t*‐test or Mann–Whitney–Wilcoxon test compared quantitative variables.

^b^ 2 missing data.

^c^ 1 missing data.

### 3.4 Factors Associated With Personal‐Social Skills at 2 Years

Using binary logistic regression, three factors were associated with a social ASQ sub‐score below two standard deviations. These factors were an increase in ΔHRV at 34 weeks' gestational age, the presence of neurological lesions, and smoking during pregnancy (Table 3). Due to the small number of infants with abnormal social scores, multivariate analysis was not performed. The complete results of the univariate analyses are presented in Table S4.

**TABLE 3:** Significant univariate associations between perinatal variables and personal‐social skills evaluated by the ASQ at 2 years.

Data	Category	*N* (%)^a^	Abnormal ASQ score^a^	OR [95% CI]	*p*
Personal–social (*n* = 87)					
Tobacco	No	81 (93.1)	4 (66.7)	1	< 0.05
	Yes	6 (6.9)	2 (33.3)	9.57 [1.40; 65.55]	
Neurological lesions	No	68 (78.2)	2 (33.3)	1	< 0.05
	Yes	19 (21.8)	4 (66.7)	7.72 [1.46; 40.88]	
ΔHRV at 34 weeks of PMA^b^ (1 week variation)		87 (100)	6 (100)	2.05 [1.02; 4.14]	< 0.05

Abbreviations: ASQ, Ages and Stages Questionnaire, third edition; OR [95% CI], odds ratio [95% confidence interval].

^a^ Data are presented as *N* (%).

^b^ HRV, heart rate variability; a binary logistic regression was employed to assess the relationship between individual‐social score evaluated by ASQ at 2 years and clinical features.

## 4 Discussion

This study tested the use of a machine learning model designed from a population of uncomplicated newborns to quantify neonatal maturation of the central autonomic network. With the model used, delayed maturation, early and non‐invasively estimated by ΔHRV, depended on gestational age and neonatal complications. The study results also suggest that this quantification of delayed maturation could be a useful biomarker for neurodevelopment.

We observed that the delay in maturation, measured by, was approximately 1 to 3 weeks at 34 weeks of PMA. This was expected because there is a delay in maturation associated with preterm birth [13, 14]. With the model used, ΔHRV increased with postnatal age during hospitalisation in the neonatal unit. This can be explained by the population used to train the model, which was not limited to very preterm infants but included the entire range of gestational ages.

The ΔHRV significantly increased with low GA and low Apgar score at 5 min, and was associated with BPD and PDA. Increased ΔHRV was also associated with alteration in social subscale scores of ASQ at 2 years of age. Its assessment could contribute to the evaluation of neurodevelopmental risk.

In this prospective multicenter study, only newborn infants with an optimal maturation trajectory were included in the training model. This differed from the machine learning model used by Iyer et al. to study autonomic maturation [15]. In that study, they used a heterogeneous training population of 67 extremely preterm infants recruited at the Medical University of Vienna, including infants with neonatal complications. This difference in strategy could explain why Iyer et al. did not find a significant association between maturation delay and perinatal complications or neurodevelopmental outcomes.

BPD is known to be associated with overall neurodevelopmental disabilities at 5 years of age [27]. To our knowledge, this is the first time that BPD has been associated with delayed maturation of the central autonomic network, as assessed by HRV in very preterm infants. This could be used as an early marker to monitor the impact of BPD on neurological functional maturation.

The impact of PDA and its treatment on neurological maturation remains debated. While hemodynamically significant PDA is known to increase vagal‐mediated HRV, it has not been linked to delayed autonomic maturation [28]. Our findings support the trend of maturational delay in infants with PDA, as reported by Iyer et al. [15].

We cannot draw conclusions about the impact of neurological lesions on central autonomic network maturation from our study. Iyer et al. reported that the relationship between PMA and functional autonomic age was not confounded by the presence of IVH (*p* = 0.63) [15]. In our study, there was a non‐significant trend toward increasing ΔHRV according to the severity and type of neurological lesion (*p* = 0.07). However, it should be noted that the neurological lesions observed were heterogeneous, with a low incidence of severe lesions, and that the statistical power was insufficient for each of them. Furthermore, it is possible that the model used was unable to recognise the specific HRV profiles associated with each perinatal brain injury [29].

Increased ΔHRV at 34 weeks of PMA was associated with the risk of having an abnormal personal‐social sub‐score at 2 years. We found a two‐fold increase in the risk of scoring below the threshold for this domain for each additional week of ΔHRV. However, increased ΔHRV was not associated with the other ASQ sub‐scores. Several studies have suggested a correlation between HRV and neurodevelopment [18], with a potential predictive value of HRV in neurodevelopmental alterations [19]. Our results seem to point in this direction, but limitations need to be taken into account. The response rate of 66% was similar to that reported in other larger multicenter studies that used this assessment method [2]. However, we observed that the populations with and without ASQ data available at 2 years were not strictly comparable. The differences involved variables that have a known impact on neurodevelopment, such as maternal age, GA, and the presence of neurological lesions. This could have contributed to the relatively low proportion of preterm infants with abnormal ASQ scores at age two. We observed that, except for the communication sub‐score, the proportion of scores below the threshold was low in our study. In our population, an ASQ score below the threshold was found for 25% of the infants at 2 years of age. In the French cohort EPIPAGE 2, which studied neurodevelopmental outcomes for 2506 preterm infants in 2011, this was around 40% for similar GA [2]. Other potential explanations for the low proportion of preterm infants with abnormal ASQ scores are hypothetical. We believe that the good quality of post‐discharge intervention programmes in place at the centres involved in this study may have contributed [30, 31]. In all centres involved in this study, preterm infants born before 30 weeks GA benefited from systematic monitoring and specialised neurodevelopmental interventions. This could have attenuated the association between increased ΔHRV at 34 weeks of PMA and neurodevelopment at 2 years. Moreover, the use of the ASQ as a self‐administered questionnaire could be affected by reporting and social bias. Despite its limitations, this method has proven reliable for assessing neurological development and has been widely used in preventive and curative healthcare programmes [32].

The association between ΔHRV and impaired social skills aligns with the known functional architecture of brain networks. This association was not unexpected, as the central autonomic nervous system is involved in the complex brain network that determines the substrate of social behaviour. A similar link between HRV and social skills was observed in Cainelli et al.'s study of children aged 6–9, with or without neuropsychiatric risk factors from perinatal insults, including prematurity [20]. They also found an association between HRV and social skills, assessed using the developmental neuropsychological assessment named NEPSY‐II.

Our study, underpowered due to the small number of infants with abnormal outcomes, cannot draw definitive conclusions about the predictive value of HRV for neurodevelopmental risk. While ΔHRV was associated to delays in social skills, it did not significantly correlate with other neurodevelopmental outcomes. This limits its broader predictive applicability and suggests that larger cohorts and further validation are needed. Increased ΔHRV at 34 weeks of PMA was one of three variables significantly associated with abnormal social skills at 2 years. This suggests that ΔHRV at 34 weeks of PMA could be included in future models designed to assess the risk of neurodevelopmental disorders. The finding that smoking during pregnancy was significantly associated with an abnormal social individual sub‐score is an interesting secondary result of our study. The effects of prenatal tobacco use on neurodevelopment are not yet well established, with results varying across studies [33, 34, 35]. It is plausible that the direct effect of tobacco on the fetus could influence neurological development. It is also possible that smoking was an indicator of a poor postnatal environment, involving low socio‐economic status of the parents and/or a lack of positive parenting.

## 5 Strength and Limitations

This prospective and multicenter study used two different populations to train and then test the machine learning model. We believe that the methodology used, the number of preterm infants included, and the results presented allow us to consider monitoring of ΔHRV as potentially useful. The main limitations of this study are related to the population studied. The robustness and generalisability of the proposed approach could be improved by increasing the diversity of newborns included in the model training phase. Larger test panels are also needed to validate ΔHRV as a useful early biomarker of neurodevelopment.

## 6 Conclusion

In the very preterm infants studied, ΔHRV evaluated through the proposed ensemble machine learning approach was influenced by clinical events occurring during initial hospitalisation in neonatology. The monitoring of ΔHRV could help clinicians to early identify abnormal individual maturation trajectories. These deviations were associated with abnormal neurodevelopment in our study. Therefore, we believe that ΔHRV could be tested as a biomarker to facilitate the implementation of early individualised interventions aimed at preventing neurodevelopmental abnormalities.

## Author Contributions

Léa Bonneau and Patrick Pladys conceptualised the design of the study and overall motivations of the study. Léa Bonneau, Patrick Pladys, Guy Carraut and Maxime Esvan processed, analysed, and interpreted the data. Léa Bonneau and Patrick Pladys wrote and edited the draft of the manuscript. Cyril Flamant, Maxime Esvan, Jean Michel Roué, Géraldine Favrais, Géraldine Gascoin, Sandy Cabon, Fabienne Porée, and Guy Carrault contributed to editing the manuscript and provided critical revisions of the manuscript.

## Funding

The results incorporated in this publication received funding from the European Union's Horizon 2020 research and innovation programme under grant agreement no. 689260 (Digi‐NewB project).

## Conflicts of Interest

The authors declare no conflicts of interest.

## Supporting information


**Appendix S1:** apa70462‐sup‐0001‐AppendixS1.docx.

## Data Availability

The data that support the findings of this study are available from the corresponding author upon reasonable request.
