# Supplementary material for: Early Maturation of Heart Rate Variability in Very Preterm Infants Depends on Neonatal Factors and Is Associated With Neurodevelopmental Risk
Source: Acta Paediatr. 2026 Feb 12;115(6):1191–201. doi: 10.1111/apa.70462 (PMC13159783; doi:10.1111/apa.70462)
Supplement: Supplementary file 1 — Appendix S1: apa70462‐sup‐0001‐AppendixS1.docx. [file APA-115-1191-s001.docx]

**Table S1. Exclusion criteria for the reference group (healthy newborns used to train the HRV model).**

| Maternal cardiac or neurological treatment |
| --- |
| Maternal drug abuse |
| Chest compression for resuscitation at birth |
| Presence of any of the following neurological lesions: grade 2, 3 or 4 intraventricular hemorrhage; white matter injury; hypoxic-ischemic encephalopathy; any abnormal brain imaging at term post-menstrual age |
| Early- and late-onset sepsis with a duration of anti-infectious therapy of more than five days |
| Necrotizing enterocolitis |
| Bronchopulmonary dysplasia |
| Severe malformations |
| Birth weight lower than the Fenton’s 10th centile for their GA |

**Table S2: Comparison of included and excluded patients**

|  | Included patient ^a^  (n=132) | Excluded patients^a^  (n=38) | P value |
| --- | --- | --- | --- |
| Maternal and antenatal characteristics | |  |  |
| Maternal age at birth (years) | 32.0 (27.0; 35.0) | 30.5 (26.0; 35.0) | 0.534 |
| Complication of pregnancy  Hypertension  Choriomniotitis  Premature rupture of membranes  Diabetes type 2 or gestational | 25 (18.9)  34 (25.8)  43 (32.6)  12 (9.1) | 9 (23.7)  6 (15.8)  14 (36.8)  4 (10.5) | 0.678  0.310  0.767  0.757 |
| Betamethasone exposure | 125 (94.7) | 33 (86.8) | 0.142 |
| Magnesium exposure | 106 (80.3) | 32 (84.2) | 0.758 |
| Active smocking | 14 (10.6) | 8 (21) | 0.103 |
| Birth characteristics | |  |  |
| Gestational Age (weeks) | 27.3 (26.1; 28.7) | 28.0 (27.0; 28.9) | 0.030 |
| Male | 68 (51.5) | 16 (42.1) | 0.401 |
| Weight (percentile for Gestational age) | 53.0 (23.5; 73.0) | 37.0 (17.5; 54.7) | 0.032 |
| Cesarian section | 73 (55.3) | 30 (78.9) | 0.014 |
| Apgar score at 5 min^b^ | 8.0 (7.0; 10.0) | 8.0 (7.0; 9.0) | 0.894 |
| Resuscitation at birth  Ventilation  Chest compression | 126 (95.5)  10 (7.6) | 38 (100)  2 (5.2) | 0.339  1.0 |
| Complication in hospitalisation | |  |  |
| Neurological lesion | 37 (28) | 5 (13.1) | 0.086 |
| Bronchopulmonary dysplasia | 45 (34.1) | 6 (15.8) | 0.049 |
| Patent ductus arteriosus | 86 (65.2) | 17 (44.7) | 0.037 |

^a^ Data are presented as n (%) for categorical variables and median (first quartile; third quartile) for continuous variables; ^b^ 3 missing data

**Table S3 : Comparison of patients with and without Ages and Stages Questionnaire (ASQ) assessment at 2 years**

|  | Patient with ASQ ^a^  (n=87) | Patient without ASQ ^a^  (n=45) | P value |
| --- | --- | --- | --- |
| Maternal and antenatal characteristics | | | |
| Maternal age (years) | 32.0 (29.0; 36.0) | 27.0 (25.0; 34.0) | 0.020 |
| Complication of pregnancy  Hypertension  Choriomniotitis  Premature rupture of membranes  Diabetes type 2 or gestational | 17 (19.5)  27 (31.0)  28 (32.2)  5 (5.7) | 8 (17.8)  7 (15.6)  15 (33.3)  7 (15.6) | 0.806  0.053  0.893  0.106 |
| Active smocking | 6 (6.9) | 8 (17.8) | 0.073 |
| Betamethasone exposure | 83 (95.4) | 42 (93.3) | 0.689 |
| Magnesium exposure | 72 (82.8) | 34 (75.6) | 0.324 |
| Birth characteristics | | | |
| Gestational age (weeks) | 27.6 (26.4; 28.7) | 26.9 (25.3; 28.3) | 0.039 |
| Male | 44 (50.6) | 24 (53.3) | 0.763 |
| Weight (percentil for gestational age) | 55.0 (26.0; 75.0) | 50.0 (22.0; 72.0) | 0.522 |
| Delivery mode  Vaginal delivery  Cesarian section | 35 (40.2)  52 (59.8) | 24 (53.3)  21 (46.7) | 0.151 |
| Apgar score at 5 min^b^ | 9.0 (7.0; 10.0) | 8.0 (7.0; 9.0) | 0.449 |
| Resuscitation at birth  Ventilation  Chest compression | 82 (94.3)  6 (6.9) | 44 (97.8)  4 (8.9) | 0.663  0.734 |
| Complication in hospitalisation | | | |
| Neurological lesion | 19 (21.8) | 18 (40) | 0.027 |
| Bronchopulmonary dysplasia | 29 (33.3) | 16 (36.5) | 0.798 |
| Patent ductus arteriosus | 53 (60.9) | 33 (73.3) | 0.155 |

^a^ Data are presented as n (%) for categorical variables and (first quartile; third quartile) for continuous variables; ^b^ 3 missing data

**Table S4. Univariate analyses for association between perinatal variables and social-individual skill evaluated by Age and Stages Questionnaire (ASQ) at 2 years**

| **Data** | **Category** | **N (%)^a^** | **Abnormal ASQ score^a^** | **OR [IC95%]^b^** | **p value** |
| --- | --- | --- | --- | --- | --- |
| **Maternal and antenatal variables** | | | | | |
| Arterial hypertension | NO  YES | 70 (80.5)  17 (19.5) | 5 (83.3)  1 (16.7) | 1.08 [0.16 ; 7.46]  2 | *0,936* |
| Chorioamniotitis | NO  YES | 60 (69.0)  27 (31.0) | 4 (66.7)  2 (33.3) | 1  1.23 [0.24 ; 6.34] | *0,803* |
| Premature rupture of membranes | NO  YES | 59 (67.8)  28 (32.2) | 3 (50.0)  3 (50.0) | 1  2.22 [0.46 ; 10.69] | *0,322* |
| Diabetes (type 2 or gestational) | NO  YES | 82 (94.3)  5 (5.7) | 6 (100)  0 (0) | 1  1.07 [0.04 ; 28.21] | *0,967* |
| Betamethasone exposure | NO  YES | 4 (4.6)  83 (95.4) | 0 (0)  6 (100) | 1  0.75 [0.03 ; 21.81] | *0,869* |
| Magnesium exposure | NO  YES | 15 (17.2)  72 (82.8) | 0 (0)  6 (100) | 1  3.03 [0.15 ; 62.05] | *0,471* |
| Active smocking | NO  YES | 81 (93.1)  6 (6.9) | 4 (66.7)  2 (33.3) | 1  9.57 [1.40 ; 65.55] | *0,021* |
| **Birth variables** | | | | | |
| Gestational age  (weeks) | <26  26-28  ≥ 28 | 35 (40.2)  15 (17.2)  37 (42.5) | 2 (33.3)  3 (50.0)  1 (16.7) | 1.82 [0.22 ; 14.92]  6.81 [0.87 ; 53.38]  1 | *0,139* |
| Infant’s sex | Female  Male | 43 (49.4)  44 (50.6) | 1 (16.7)  5 (83.3) | 1  3.95 [0.60 ; 25.81] | *0,152* |
| Weight (percentil for gestational age) | <25  25-50  50-75  ≥75 | 21 (24.1)  18 (20.7)  26 (29.9)  22 (25.3) | 1 (16.7)  1 (16.7)  2 (33.3)  2 (33.3) | 1  1.17 [0.11 ; 13.06]  1.39 [0.16 ; 12.01]  1.67 [0.19 ; 14.51] | *0,970* |
| Delivery mode | Vaginal  Cesarean | 35 (40.2)  52 (59.8) | 4 (66.7)  2 (33.3) | 1  0.35 [0.07 ; 1.76] | *0,201* |
| Apgar score at 5 minutes^c^ | <7  ≥7 | 19 (21.8)  65 (74.7) | 2 (33.3)  4 (66.7) | 1  1.95 [0.37 ; 10.34] | *0,431* |
| Ventilation | NO  YES | 5 (5.7)  82 (94.3) | 0 (0)  6 (100) | 1  0.93 [0.04 ; 24.61] | *0,967* |
| Chest compression | NO  YES | 81 (93.1)  6 (6.9) | 5 (83.3)  1 (16.7) | 1  3.79 [0.45 ; 32.10] | *0,221* |
| **Complication in hospitalisation** | | | | | |
| Bronchopulmonary dysplasia | NO  YES | 58 (66.7)  29 (33.3) | 4 (66.7)  2 (33.3) | 1  1.10 [0.21 ; 5.65] | 0,908 |
| Neurological lesions | NO  YES | 68 (78.2)  19 (21.8) | 2 (33.3)  4 (66.7) | 1  7.72 [1.46 ; 40.88] | *0,016* |
| Patent ductus arteriosus | NO  YES | 34 (39.0)  53 (61.0) | 0 (0)  6 (100) | 1  9.45 [0.49 ; 180.74] | *0,135* |
| ΔHRV ^d^ |  | 87 (100) | 6 (100) | 2.05 [1.02 ; 4.14] | *0,044* |

^a^ Data are presented as N(%); ^b^ OR: Odds Ratio; IC: Confidence Interval; ^c^ 3 missing data; ^d^ 1 week variation
